# Peanut sensitization in a group of allergic Egyptian children

**DOI:** 10.1186/1710-1492-7-11

**Published:** 2011-05-31

**Authors:** Elham Hossny, Ghada Gad, Abeer Shehab, Amgad El-Haddad

**Affiliations:** 1 Departments of Pediatrics, Ain Shams University, Ramses Street, Cairo 11566, Egypt; 2 Department of Clinical Pathology, Ain Shams University, Ramses Street, Cairo 11566, Egypt; 3 Allergy/Immunology Unit, VACSERA, Cairo 12311, Egypt

## Abstract

**Background:**

There are no published data on peanut sensitization in Egypt and the problem of peanut allergy seems underestimated. We sought to screen for peanut sensitization in a group of atopic Egyptian children in relation to their phenotypic manifestations.

**Methods:**

We consecutively enrolled 100 allergic children; 2-10 years old (mean 6.5 yr). The study measurements included clinical evaluation for site of allergy, possible precipitating factors, consumption of peanuts (starting age and last consumption), duration of breast feeding, current treatment, and family history of allergy as well as skin prick testing with a commercial peanut extract, and serum peanut specific and total IgE estimation. Children who were found sensitized to peanuts were subjected to an open oral peanut challenge test taking all necessary precautions.

**Results:**

Seven subjects (7%) were sensitized and three out of six of them had positive oral challenge denoting allergy to peanuts. The sensitization rates did not vary significantly with gender, age, family history of allergy, breast feeding duration, clinical form of allergy, serum total IgE, or absolute eosinophil count. All peanut sensitive subjects had skin with or without respiratory allergy.

**Conclusions:**

Peanut allergy does not seem to be rare in atopic children in Egypt. Skin prick and specific IgE testing are effective screening tools to determine candidates for peanut oral challenging. Wider scale multicenter population-based studies are needed to assess the prevalence of peanut allergy and its clinical correlates in our country.

## Introduction

The prevalence of peanut allergy is not sufficiently studied in many developing countries including Egypt. Peanut allergy was estimated to affect 0.8% of children and 0.6% of adults in the US, showing a twofold increase over a 5-year period [1,2]. A recent 11 year follow up survey showed that the prevalence of peanut allergy in children in the US in 2008 was 1.4% compared to 0.8% in 2002 and 0.4% in 1997 [3]. In the United Kingdom, the total estimate for clinical peanut allergy was1.5% of 3-4 year-old children [4]. Relevant studies estimated the prevalence of peanut allergy to be 1.34% among primary school children in a Canadian province [5] and 1.15% among 3 year olds in the Australian capital territory with a trend for rise in prevalence between 1997 and 2005 [6]. Peanut was the third most common sensitizing allergen in an Asian community especially in young atopic children with multiple food hypersensitivities and a family history of atopic dermatitis [7].

Studies to address the reasons for increased prevalence and persistence of food allergies, focusing primarily on peanut, have included the hygiene hypothesis; changes in the components of the diet, including antioxidants, fats, and nutrients, such as vitamin D; the use of antacids, resulting in exposure to more intact protein; food processing, such as for peanut roasting and emulsification to produce peanut butter compared with fried or boiled peanut; and extensive delay of oral exposure, thus increasing topical (possibly sensitizing) rather than oral (possibly tolerizing) exposure to food allergens [8,9].

The evaluation of a child with suspected allergy to peanut should include a careful history taking, skin-prick testing (SPT), measurement of serum-specific IgE, and, confirmation by an oral food challenge [10,11]. The prevalence of confirmed food allergy based on confirmatory tests is lower than perceived allergy which is based on self report [12,13]. Diagnostic cut-off values for SPT and specific IgE results have improved the diagnosis of food allergy and thereby reduced the need to perform oral food challenges [14]. Overall, a negative peanut SPT has a negative predictive value of more than 95% [15]. The positive predictive value, however, is significantly lower, reaching only 60% in patients with a convincing history of an allergic reaction [16]. Published values on positive SPT and specific IgE values vary from one series to another depending on several factors [12,17-20]. Cut-off values do not always have general acceptability and sometimes need to be individualized in the context of clinical impression [21].

There is an impression that peanut allergy is uncommon in Egypt and there are no published data on its incidence or prevalence. We sought to investigate the frequency of peanut sensitization and allergy in a group of atopic Egyptian infants and children in a pilot attempt to uncover its importance as an allergen in our country.

## Methods

### Study Population

This cross sectional study comprised 100 children diagnosed to have allergic diseases. They were enrolled consecutively after getting informed oral consent was obtained from the parents or care-givers. The study protocol gained approval from the local ethics committee.

Inclusion criteria:

-	Age at enrollment between one and 18 years.

-	A physician made diagnosis of allergic diseases including asthma, allergic rhinitis, urticaria, and eczema.

Exclusion criteria:

-	Patients who cannot stop antihistamine therapy.

-	Extensive skin lesions, scars, positive dermatographism, and very dark skin.

-	Treatment with systemic corticosteroids for more than 7 days.

-	Other chronic or debilitating illness.

### Study Measurements

All patients included in the study were subjected to the following:

#### 1. Clinical evaluation

Detailed history was taken for the possible precipitating factors, peanut consumption (starting age and last consumption), duration of breast feeding, and family history of allergy. Patients were subjected to a general clinical examination, as well as chest, skin, and ENT examination to verify the diagnosis.

#### 2. Laboratory investigations

*Serum total *IgE was measured by enzyme linked immunosorbent assay (Genzyme Diagnostics, Medix Biotech Inc, San Carlos, CA, USA). A serum IgE level was considered elevated if it exceeded the highest reference value for age [22]. The value used in correlation analysis was the percentage from the highest normal value for age (patient's actual value/highest normal value for age multiplied by 100)

*Peanut Specific IgE *was measured in children with positive peanut SPT results using the CLA allergen-specific IgE Assay according to the manufacturer's instructions (Hitachi Chemical Diagnostics, Mountain View, California 94043, USA). The concentration of ≥ 15 kUA/L was considered positive.

*Complete blood counting *was done using an automated cell counter (Coulter MicroDiff 18, Fullerton, CA, USA) and manual differential.

#### 3. Skin prick testing

Skin prick test (SPT) was performed for each patient using a commercial peanut allergen extract, positive histamine control, and negative control (Omega Laboratories, Montréal, Canada). First generation short-acting antihistamines were avoided for at least 72 hours and second generation antihistamines were avoided for at least 5 days before testing. The test sites were marked and labeled at least three cm apart to avoid the overlapping of positive skin reactions. The marked site was dropped by the allergen and gently pricked by sterile skin test lancet. Positive and negative control solutions were similarly applied. The patient waited for at least 20 minutes before interpretation of the results. Largest and orthogonal diameters of any resultant wheal and flare were measured. A wheal diameter of 8 mm or greater was considered positive.

#### 4. Oral food challenge (OFC)

Children with proven peanut sensitization were subjected to open oral peanut challenges under close medical observation taking all the precautions needed to treat anaphylaxis. A second informed consent was obtained from the parents or care-givers prior to the challenge. Children were given gradually increasing amounts of roasted peanuts at 30 min intervals and symptoms and physical signs were closely monitored. The total dose of peanut before considering that the challenge was negative was 15 grams of roasted whole peanuts. An open feeding of a larger portion (age-appropriate serving) followed negative challenges and then children were kept under observation for 2 more hours. The cases with negative challenges were supplied with a contact phone number to report any reactions that might develop within the next 24 hours while cases with positive challenge were treated and kept in hospital for 12-24 hours under observation.

### Statistical analysis

Data were analyzed by a standard computer program (SPSS version 13 for Windows, Chicago IL, USA). The mean, standard deviation (SD), median, and interquartile (IQ) range presented the descriptive data. Groups were compared using the students t- test for parametric and the Kruskal-Wallis and Mann- Whitney Z tests for non- parametric data. Fisher's Exact and Chi square (X^2^) tests were used for comparison of categorical data. Pearson and Spearman coefficient tests were used to correlate the numeric data. For all tests, p values less than 0.05 were considered statistically significant.

## Results

The studied sample comprised 55 boys and 45 girls. Their ages ranged between two and 10 years [median (IQR) = 6.28 (4.0); mean (SD) = 6.51 (2.35) years]. The duration of exclusive breast feeding ranged from 3 to 8 months [median (IQR) = 6.00 (2.00); mean (SD) = 5.42 (1.32) months] and the age of stoppage of breast feeding ranged from 9 to 24 months [median (IQR) = 16.00 (7.00); mean (SD) = 16.30 (4.26) months]. None of the subjects gave a history suggestive of peanut allergy and all of them started consuming roasted peanuts before the age of two. The duration since last peanut consumption ranged between one and 15 days [median (IQR) = 4.00 (3.00); mean (SD) = 4.63 (8.94) days]. The diagnoses included bronchial asthma in 63 children, urticaria in 57, allergic rhinitis in 22, atopic dermatitis in five, and history of anaphylaxis due to unknown cause in one patient. Fifty six children had one, 40 had two, and four had three of the aforementioned allergic diseases.

Skin prick testing with peanut extract gave positive results (wheal diameter ≥ 8 mm) in seven children (7%). The specific IgE results of these children confirmed sensitization [range = 17 - 24; median (IQR) = 21.0 (4.00); mean (SD) = 20.9 (2.41); 95% CI = 16.9 - 24.5 kUA/L]. Six out of the 7 peanut sensitized patients consented for an open oral challenge with roasted whole peanuts. Three out of the six children showed immediate allergic manifestations after consumption (two developed urticaria and respiratory manifestations and one developed urticaria only) and were thus proven to have allergy to peanut. The remaining three developed no symptoms or signs upon oral challenge with peanuts (Table 1). Neither the positive nor negative OFC cases developed late phase reactions to peanut. Two of the peanut allergic children were brothers (Table 1; patients 1 and 2). They had bronchial asthma and attacks of urticaria and the elder one also suffered from allergic rhinitis. The peanut specific IgE levels could not be correlated to any of the clinical or laboratory data of the sensitized children. Children with positive oral challenge were statistically comparable to those with negative results as far as their clinical and laboratory data are concerned.

**Table 1:** Clinical and laboratory data of the peanut sensitized children

n	Sex	Age (yr)	FH of allergy	BF only (mo)	BF stopped (mo)	Allergy manifestations (target organs)	Other food allergy	Wheal (mm)	Flare (mm)	Total IgE (kUA/l)	Specific IgE (kUA/l)	**AEC (/mm**^**3**^**)**	OFC test
1	M	9	+	6	15	AR - BA - Urticaria	-	10	20	221	23	300	+

2	M	7	+	4	18	BA - Urticaria	-	9	18	76	20	120	+

3	M	9	+	6	15	BA - Urticaria	-	9	18	130	24	100	+

4	F	9	+	5	12	AD - Urticaria	-	9	18	115	22	240	-

5	M	9	+	6	12	AR - BA - Urticaria	-	9	19	266	19	300	-

6	M	5	+	4	16	AD - Anaphylaxis	-	8	16	85	17	80	ND

7	F	5	+	7	13	Urticaria	+	9	20	212	21	70	-

AD: atopic dermatitis; AR: allergic rhinitis; AEC: absolute eosinophil count; BA: bronchial asthma; BF: breast feeding; F: female; FH: family history; M: male; ND: not done; OFC: oral food challenge.

Seventy percent of the studied sample gave a positive family history of allergy with no statistically significant relation to peanut sensitization. However, the 7 children sensitized to peanut had positive family history of allergic diseases. None of the subjects gave a history suggestive of peanut allergy in the family. Peanut SPT results did not vary according to gender. Positive results were obtained in 5 out of 55 boys and 2 out of 45 girls. Peanut sensitization rates were not influenced by the duration of exclusive breast feeding, age at complete weaning from the breast, last peanut consumption, serum total IgE level, or peripheral blood eosinophil count (Table 2).

**Table 2:** Variation of some clinical and laboratory data according to peanut sensitization*

Variable	Peanut + (n = 7)	Peanut - (n = 93)	Test	p value
Age (yr) - median (IQR)	9.0 (4.00)	6.0 (4.75)	Z = - 0.225	0.221

Gender (male/female)	05/02	50/43	X^2 ^= 0.821	0.365

Positive family history of allergy (%)	100%	75.3%	X^2 ^= 2.248	0.134

Exclusive BF duration (mo) - median (IQR)	6.0 (2.0)	6.0 (2.0)	Z = - 0.071	0.943

Age of BF stoppage (mo) - median (IQR)	15.0 (4.0)	16.0 (6.0)	Z = - 1.211	0.226

Last peanut consumption (days) - median (IQR)	5.0 (2.0)	4.0 (3.0)	Z = -0.807	0.420

Last antihistamine consumption (days) - median (IQR)	6.0 (3.0)	5.0 (4.0)	Z = -1.096	0.273

AEC (cells/mm^3^) - median (IQR)	120.0 (220.0)	90.0 (62.5)	Z = - 1.935	0.053

Total IgE (kUA/L) - median (IQR)	130.0 (136.0)	113.0 (50.0)	Z = - 1.008	0.313

AEC: absolute eosinophil count; BF: breast feeding; IQR: interquartile range; Peanut +: peanut sensitive; Peanut -: not sensitized to peanut.

* Peanut sensitization: SPT wheal diameter ≥ 8 and specific IgE ≥ 15 kUA/L

The SPT results were not influenced by the target organ affected whether respiratory or cutaneous. Also, peanut sensitization did not vary according to the number of target organs affected in the studied sample (X^2 ^= 2.714; p = 0.257). Ten children had confirmed allergy to other foods (egg allergy in two, fish in three, cow milk in two, sesame in one, banana in one, and prunes in one); 9 of them were not peanut sensitized while one was sensitized to peanut and allergic to bananas. The relation between peanut sensitization rates and the presence of other food allergies did not reach statistical significance (X^2 ^= 0.154; p = 0.695).

The wheal diameter of the peanut sensitized children was not correlated to age at complete weaning from the breast, days since last peanut consumption, days since last antihistamine consumption, serum total IgE, serum specific IgE, peripheral blood eosinophil count, or histamine wheal diameter.

## Discussion

The frequency of peanut sensitization in our series was 7%. The sensitized children were subjected to open oral challenges except for one child whose parents did not consent to the challenge due to a past history of anaphylaxis of unknown etiology. Only three patients were proven to be allergic to peanut by oral challenge giving a 3% rate of peanut allergy in the studied sample. The studied sample comprised children with physician-diagnosed allergy and therefore does not represent the general population. The Peanut specific IgE was only estimated in the 7 children with positive SPT for confirmation of sensitization. It would be worthwhile to estimate its expression in children with negative SPT. However, this was not among the objectives of the current study.

Population based data from some other parts of the world show different rates of sensitization ranging between 3.3% in England [4] and 4.6% among children from the Netherlands [23]. Cross sectional random telephone surveys revealed self reported peanut allergy in 0.6-1.4% in the US [2,3], 0.93-1% in Canada, and 1.5% in the UK [12]. The prevalence of peanut allergy is relatively low in Asian children (0.43% - 0.64%) [24].

We considered a SPT wheal size of 8 mm and specific IgE level of 15 kUA/L as our cut off values for predicting peanut allergy [17,25]. Nevertheless, only half of the sensitized children had clinical reactions upon oral challenge. We performed an oral challenge despite the fact that our cut off values may diagnose peanut allergy because none of the sensitized subjects gave a history suggestive of peanut allergy. It seems that the cut off values should be tailored to the levels of consumption in different geographical locations. A recent study from the UK reported a 22.4% prevalence of clinical peanut allergy among sensitized subjects. The authors used the same wheal diameter and specific IgE cut off values that we used [25]. The published peanut specific IgE cut off values are variable ranging between 5 up to 57 kUA/L [26,27]. A wheal diameter of 16 mm was considered to have a positive predictive value of 100% for allergy to peanuts [27].

Although a prior probability from the history is an important starting point in the diagnosis of peanut allergy, none of our subjects gave a history suggestive of peanut allergy and all of them started consuming roasted peanuts before the age of 2 years. Roasted peanut is a popular snack in Egypt and its allergy is not a public concern due to underestimation of its significance even from the health care workers' point of view. The history was reported to be notoriously poor (approximately 30% verified) in identifying causal foods for chronic disorders such as atopic dermatitis [16]. The early peanut consumption by children does not seem to be a risk factor for peanut allergy and was postulated to be even protective [28].

There was no significant relation between the duration of breast feeding and peanut sensitization in the current study. A relevant study noted that neither the maternal peanut consumption during pregnancy and lactation nor the duration of breast feeding was associated with the development of peanut allergy [29]. On the other hand, a recent case-control study assumed that exposure to peanut allergens in utero or through breast milk may increase the risk of developing peanut allergy [30].

A family history of atopy, especially of food allergy, is a good screening test to identify an individual at risk of food allergy and the rate of allergy in a sibling of an allergic person is known to be higher than the rate in the general population. Although we could not demonstrate a statistically significant relation between the family history of allergy and peanut sensitization, all peanut sensitive children came from atopic families. Also, two of the peanut allergic children were brothers. The current study did not demonstrate a significant gender difference in peanut sensitization but the boys outnumbered girls in the whole sample. Higher prevalence of peanut allergy among male children was previously reported [31].

The peanut sensitization in the present study did not vary according to the site of allergy. However, the 7 peanut sensitized children had physician diagnosed skin allergy combined with respiratory allergy in five. A relevant study on Asian children revealed that most (89.5%) of first reactions featured skin changes and that respiratory and GI symptoms did not occur as the sole manifestation [32]. One child with positive oral challenge in our series had allergy in one target-organ (skin) and a past history of one attack of unexplained anaphylaxis and two children had symptoms in more than one system (skin and respiratory). According to a voluntary registry in the Isle of Wight, about half of all children with peanut allergy had allergic manifestations in one target-organ system, 30% in two, 10% - 15% in three, and 1% had symptoms in four systems [33].

Self-reported food allergy is an independent risk factor for potentially fatal childhood asthma [34]. Peanut allergy was reported in 28.8% of children with asthma in a recent investigation [35]. One of our peanut sensitized children was allergic to bananas. Her peanut oral challenge yielded a negative result. The girl was not latex sensitive and the parents did not consent to any further investigations. Evaluation of pollen cross reactivity would have been worthwhile [36].

From this pilot study, it seems that peanut allergy in Egypt is underestimated and that the sensitization rates are even higher. Skin prick and specific IgE testing aided by history are good screening tools to determine candidates for peanut oral food challenge. Peanut allergy can be associated with any clinical form of allergy and the causal relationship needs extensive evaluation. The conclusions are limited by the sample size and study design which targeted physician-diagnosed allergy rather than the general population. Further wider-scale population-based studies as well as a national registry are needed to be able to outline the real magnitude of peanut allergy and its clinical correlates in our country.

## Competing interests

The authors declare that they have no competing interests.

## Authors' contributions

EH put the study design and coordination, performed the statistical analysis, and drafted the manuscript. GG participated in collection of the study sample and data analysis. AS performed the total and specific IgE assay and differential blood cell counting. AE collected the study sample and performed the skin prick test. All authors read and approved the final manuscript.
